# Supplementary material for: Bayesian continual learning and forgetting in neural networks
Source: Nat Commun. 2025 Oct 30;16:9614. doi: 10.1038/s41467-025-64601-w (PMC12575836; doi:10.1038/s41467-025-64601-w)
Supplement: Supplementary file 1 — Supplementary Information [file 41467_2025_64601_MOESM1_ESM.pdf]

Supplementary information: Bayesian continual learning and forgetting in neural networks

Djohan Bonnet[1], Kellian Cottart[1], Tifenn Hirtzlin[2], Tarcisius Januel[2], Thomas Dalgaty[3], Elisa Vianello[2], and Damien Querlioz[1,*]

[1]Université Paris-Saclay, CNRS, Centre de Nanosciences et de Nanotechnologies, 91120 Palaiseau, France
[2]Université Grenoble-Alpes, CEA, LETI, 38400 Grenoble, France
[3]Université Grenoble-Alpes, CEA, LIST, 38400 Grenoble, France
[*]damien.querlioz@universite-paris-saclay.fr

Contents

Supplementary Note 1: Hessian approximation 2
Supplementary Note 2: Lemma demonstrations 3
Supplementary Note 3: Proposition demonstrations 4
Supplementary Note 4: Theorem demonstrations 9
Supplementary Note 5: Algorithm 14
Supplementary Note 6: Impact of the memory window N on the Permuted MNIST dataset 16
Supplementary Note 7: Impact of the memory window N on the CIFAR-10 and CIFAR-100 datasets 18
Supplementary Note 8: Inference time and accuracy 20
Supplementary Note 9: Comparison with Presynaptic Consolidation 20
Supplementary Note 10: Comparison with Uncertainty-guided Continual Bayesian Neural Networks (UCB) 21
Supplementary Note 11: Detailed trade-off analysis of MESU and FOO-VB Diagonal 23
Supplementary Note 12: MESU and Bayes by Backprop complexity 24

Supplementary Note 1: Hessian approximation

In the main text, we show that when MESU learns from mini-batches drawn i.i.d. from a single dataset, the standard deviations of the synapses are linked to the diagonal of the Hessian. Specifically, in the long-time limit,

$$\lim_{t\to\infty} \boldsymbol{\sigma}(t)^2 \;=\; \frac{1}{N}\,\frac{1}{H_D(\boldsymbol{\mu}_0) + \frac{1}{N\,\boldsymbol{\sigma}^2_{\text{prior}}}} \;=\; \frac{1}{\frac{1}{\boldsymbol{\sigma}_L^2} + \frac{1}{\boldsymbol{\sigma}^2_{\text{prior}}}}, \tag{1}$$

where $H_D(\boldsymbol{\mu}_0)$ denotes the diagonal of the Hessian evaluated at parameters $\boldsymbol{\mu}_0$, $\boldsymbol{\sigma}_L$ is defined through the posterior-likelihood relation (see Lemma 1 in the main text, Methods), and N is the effective memory window corresponding to the number of updates to retain.

Moreover, in the limit $N \to \infty$ (i.e. Bayesian continual learning without forgetting), the standard deviation follows

$$N \;\to\; \infty \;\Longrightarrow\; \lim_{t\to\infty} t\,\boldsymbol{\sigma}(t)^2 \;=\; \frac{1}{H_D(\boldsymbol{\mu}_0)}. \tag{2}$$

As described in the main text corresponds to the Fixed-point Operator for Online Variational Bayes (FOO-VB) Diagonal algorithm[1].

In both settings, each parameter's variance is inversely proportional to an estimate of the Hessian diagonal, indicating that $\boldsymbol{\sigma}^2$ can serve as a measure of synaptic "importance." To demonstrate these theoretical insights, we trained a network on MNIST[2] and compared the "importance" estimates from MESU, FOO-VB Diagonal, EWC[3], and SI[4] against the actual Hessian diagonal. We computed the Hessian at various points in training using `torch.autograd`. Suppl. Figs 1a and 1b confirm the relationships in Eqs. (1) and (2), respectively, with high accuracy.

Suppl. Fig. 1c also tracks the correlation between each algorithm's importance metric and the true Hessian diagonal over training. While EWC and SI give better estimates early on (around 1k updates), FOO-VB Diagonal and MESU eventually yield a closer match to the Hessian diagonal.

Methods of Supplementary Note 1

We used mini-batches of size 32 for all updates.

Network initialization. For a layer l with input dimension n_l and output dimension m_l, weights $\boldsymbol{\omega}_l$ are sampled as

$$\omega_{i,l} \;\sim\; \mathcal{U}\Big(-\frac{1}{\sqrt{n_l}}, \frac{1}{\sqrt{n_l}}\Big).$$

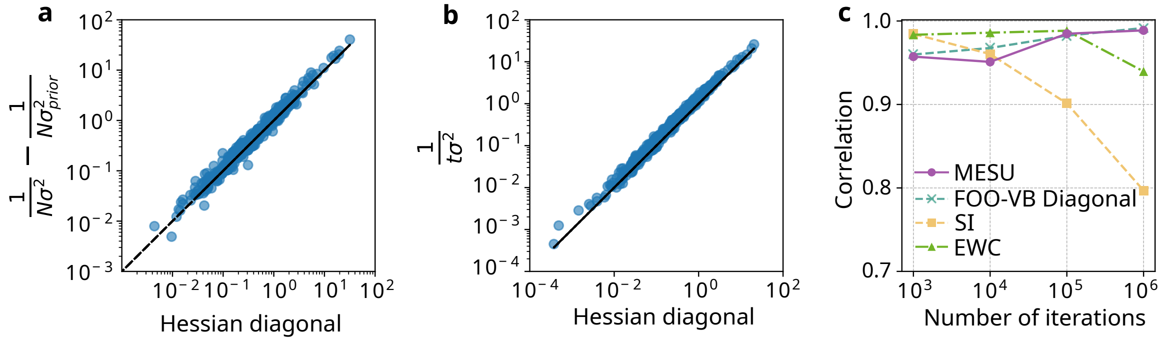

Supplementary Figure 1. Hessian approximation. a MESU's variance-based approximation vs. the actual Hessian diagonal. **b** FOO-VB Diagonal's approximation vs. the actual Hessian diagonal. **c** Correlation between each algorithm's importance metric (EWC, SI, FOO-VB Diagonal, MESU) and the Hessian diagonal over the course of training.

For MESU and FOO-VB Diagonal, the mean parameters $\mu_{i,l}$ follow a reweighted Kaiming scheme,

$$\mu_{i,l} \sim \mathcal{U}\Big(-\frac{\sqrt{2}}{\sqrt{n_l}}, \frac{\sqrt{2}}{\sqrt{n_l}}\Big),$$

and the standard deviations are initialized to $\sigma_{i,l} = \frac{1}{2\sqrt{m_l}}$.

Algorithm parameters. All algorithms use a sum-based loss reduction. Suppl. Table 1 details the specific hyper-parameters.

Algorithm	Parameter	Value
**SI**	Learning Rate α	0.0001
**EWC**	Learning Rate α	0.0001
**MESU**	Memory window N	100,000
	Learning Rate α_μ	1
	Learning Rate α_σ	1
	Prior Mean μ_p	0
	Prior Std Dev σ_p	1
**FOO-VB Diagonal**	Learning Rate α_μ	1
	Learning Rate α_σ	1

Supplementary Table 1. Hyper-parameter values for each algorithm of figure 1

Supplementary Note 2: Lemma demonstrations

Lemma 1 (The quadratic negative log-likelihood)**.** *Let $q_{\boldsymbol{\theta}}(\boldsymbol{\omega}) \approx p(\boldsymbol{\omega} \mid \mathcal{D})$ be a mean-field Gaussian for a Bayesian neural network, where $\boldsymbol{\theta} = (\boldsymbol{\mu}, \boldsymbol{\sigma})$ and $\boldsymbol{\omega} = \boldsymbol{\mu} + \boldsymbol{\epsilon} \cdot \boldsymbol{\sigma}$, $\boldsymbol{\epsilon} \sim \mathcal{N}(\vec{0}, \mathbf{I}_s)$. If the prior is $p(\boldsymbol{\omega}) = \mathcal{N}\big(\boldsymbol{\omega}; \boldsymbol{\mu}_{prior}, \mathrm{diag}(\boldsymbol{\sigma}^2_{prior})\big)$, then $p(\mathcal{D} \mid \boldsymbol{\omega}) \propto q_L(\boldsymbol{\omega}) = \mathcal{N}\big(\boldsymbol{\omega}; \boldsymbol{\mu}_L, \mathrm{diag}(\boldsymbol{\sigma}^2_L)\big)$, and the negative log-likelihood takes a quadratic form: $\mathcal{L}(\boldsymbol{\omega}) = \frac{\left(\boldsymbol{\omega}-\boldsymbol{\mu}_L\right)^2}{2\,\boldsymbol{\sigma}^2_L} + \frac{1}{2}\log\big(2\pi\,\boldsymbol{\sigma}^2_L\big)$, where $\frac{1}{\boldsymbol{\sigma}^2} = \frac{1}{\boldsymbol{\sigma}^2_L} + \frac{1}{\boldsymbol{\sigma}^2_{prior}}$ and*

$\boldsymbol{\mu} = \frac{\boldsymbol{\mu}_L \, \boldsymbol{\sigma}^2_{prior} + \boldsymbol{\mu}_{prior} \, \boldsymbol{\sigma}^2_L}{\boldsymbol{\sigma}^2_L + \boldsymbol{\sigma}^2_{prior}}$.

Proof. According to Bayes' rule we have $p(\boldsymbol{\omega}|\mathcal{D}) \propto p(\mathcal{D}|\boldsymbol{\omega})p(\boldsymbol{\omega})$, therefore $p(\mathcal{D}|\boldsymbol{\omega}) \propto \frac{p(\boldsymbol{\omega}|\mathcal{D})}{p(\boldsymbol{\omega})}$. Given that: $p(\boldsymbol{\omega}|\mathcal{D}) = \mathcal{N}\big(\boldsymbol{\omega}; \boldsymbol{\mu}, \mathrm{diag}(\boldsymbol{\sigma}^2)\big)$, and $p(\boldsymbol{\omega}) = \mathcal{N}\big(\boldsymbol{\omega}; \boldsymbol{\mu}_{\text{prior}}, \mathrm{diag}(\boldsymbol{\sigma}^2_{\text{prior}})\big)$, we get:

$$p(\mathcal{D}|\boldsymbol{\omega}) \propto \frac{\sqrt{2\pi\boldsymbol{\sigma}^2_{\text{prior}}}}{\sqrt{2\pi\boldsymbol{\sigma}^2}} e^{\frac{(\boldsymbol{\omega}-\boldsymbol{\mu}_{\text{prior}})^2}{2\boldsymbol{\sigma}^2_{\text{prior}}} - \frac{(\boldsymbol{\omega}-\boldsymbol{\mu})^2}{2\boldsymbol{\sigma}^2}} \propto e^{\boldsymbol{a}\boldsymbol{\omega}^2 + \boldsymbol{b}\boldsymbol{\omega}} \tag{3}$$

With $\boldsymbol{a} = -\frac{1}{2}(\frac{1}{\boldsymbol{\sigma}^2} - \frac{1}{\boldsymbol{\sigma}^2_{\text{prior}}})$, and $\boldsymbol{b} = (\frac{\boldsymbol{\mu}}{\boldsymbol{\sigma}^2} - \frac{\boldsymbol{\mu}_{\text{prior}}}{\boldsymbol{\sigma}^2_{\text{prior}}})$. For $p(\mathcal{D}|\boldsymbol{\omega})$, to be a probability distribution, it needs to be integrable with respect to $\boldsymbol{\omega}$, which implies $\forall i, a_i < 0$. Therefore, we have $\frac{1}{\sigma_i^2} > \frac{1}{\sigma^2_{\text{prior},i}}$. In that case we obtain an expression proportional to a Gaussian distribution $p(\mathcal{D}|\boldsymbol{\omega}) \propto q_{\text{L}}(\boldsymbol{\omega}) \sim \mathcal{N}(\boldsymbol{\omega}; \boldsymbol{\mu}_{\text{L}}, \boldsymbol{\sigma}^2_{\text{L}})$, and from $\boldsymbol{a} = -\frac{1}{2\boldsymbol{\sigma}^2_{\text{L}}}$, and from $\boldsymbol{b} = \frac{\boldsymbol{\mu}_{\text{L}}}{\boldsymbol{\sigma}^2_{\text{L}}}$ we deduce:

$$\frac{1}{\boldsymbol{\sigma}^2} = \frac{1}{\boldsymbol{\sigma}^2_{\text{L}}} + \frac{1}{\boldsymbol{\sigma}^2_{\text{prior}}} \tag{4}$$

$$\boldsymbol{\mu} = \frac{\boldsymbol{\mu}_{\text{L}}\boldsymbol{\sigma}^2_{\text{prior}} + \boldsymbol{\mu}_{\text{prior}}\boldsymbol{\sigma}^2_{\text{L}}}{\boldsymbol{\sigma}^2_{\text{L}} + \boldsymbol{\sigma}^2_{\text{prior}}} \tag{5}$$

Finally to obtain $\mathcal{L}(\boldsymbol{\omega}) = \frac{(\boldsymbol{\omega}-\boldsymbol{\mu}_{\text{L}})^2}{2\boldsymbol{\sigma}^2_{\text{L}}} + \frac{1}{2}\log(2\pi\boldsymbol{\sigma}^2_{\text{L}})$ we have to apply the definition of the negative log-likelihood to the density of $p(\mathcal{D}|\boldsymbol{\omega})$.

□

Lemma 2 (Second-order derivative via first-order derivative)**.** *For a mean-field Gaussian $q_{\boldsymbol{\theta}}(\boldsymbol{\omega})$ describing a Bayesian neural network, with $\boldsymbol{\omega} = \boldsymbol{\mu} + \boldsymbol{\epsilon} \cdot \boldsymbol{\sigma}$, $\boldsymbol{\epsilon} \sim \mathcal{N}(\vec{0}, \mathbf{I}_s)$, the expected Hessian of the negative log-likelihood $\mathcal{L}$ satisfies*

$$H_D(\boldsymbol{\mu}) \;=\; \mathbb{E}_{\boldsymbol{\epsilon}}\Big[\tfrac{\partial^2\mathcal{L}}{\partial\boldsymbol{\omega}^2}\Big] \;=\; \frac{1}{\boldsymbol{\sigma}}\frac{\partial\mathcal{C}}{\partial\boldsymbol{\sigma}},$$

where $\mathcal{C} = \mathbb{E}_{\boldsymbol{\epsilon}}[\mathcal{L}(\boldsymbol{\omega})]$.

Proof. By definition, the diagonal elements of the Hessian matrix with respect to $\boldsymbol{\mu}$ are: $H_D(\boldsymbol{\mu}) = \frac{\partial^2\mathcal{C}}{\partial\boldsymbol{\mu}^2}$. Given that: $\frac{\partial\mathcal{C}}{\partial\boldsymbol{\mu}} = \mathbb{E}_{\boldsymbol{\epsilon}}\left[\frac{\partial\mathcal{L}(\boldsymbol{\omega})}{\partial\boldsymbol{\omega}}\right]$, we deduce: $H_D(\boldsymbol{\mu}) = \mathbb{E}_{\boldsymbol{\epsilon}}\left[\frac{\partial^2\mathcal{L}(\boldsymbol{\omega})}{\partial\boldsymbol{\omega}^2}\right]$.

Similarly, we have: $\frac{\partial\mathcal{C}}{\partial\boldsymbol{\sigma}} = \mathbb{E}_{\boldsymbol{\epsilon}}\left[\frac{\partial\mathcal{L}(\boldsymbol{\omega})}{\partial\boldsymbol{\omega}} \times \boldsymbol{\epsilon}\right]$.

Using Stein's lemma, we obtain the following relation: $\mathbb{E}_{\boldsymbol{\omega}}\left[\frac{\partial\mathcal{L}(\boldsymbol{\omega})}{\partial\boldsymbol{\omega}} \times (\boldsymbol{\omega} - \boldsymbol{\mu})\right] = \boldsymbol{\sigma}^2\mathbb{E}_{\boldsymbol{\omega}}\left[\frac{\partial^2\mathcal{L}(\boldsymbol{\omega})}{\partial\boldsymbol{\omega}^2}\right]$. Therefore, $\mathbb{E}_{\boldsymbol{\epsilon}}\left[\frac{\partial\mathcal{L}(\boldsymbol{\omega})}{\partial\boldsymbol{\omega}} \times \boldsymbol{\epsilon}\right] = \boldsymbol{\sigma}\mathbb{E}_{\boldsymbol{\epsilon}}\left[\frac{\partial^2\mathcal{L}(\boldsymbol{\omega})}{\partial\boldsymbol{\omega}^2}\right]$.

We recognize the left term as $\frac{\partial\mathcal{C}}{\partial\boldsymbol{\sigma}}$ and conclude:

$$\boxed{H_D(\boldsymbol{\mu}) = \frac{1}{\boldsymbol{\sigma}}\frac{\partial\mathcal{C}}{\partial\boldsymbol{\sigma}}}. \tag{6}$$

□

Supplementary Note 3: Proposition demonstrations

Proposition 1 (Dynamic of standard deviations)**.** *Consider the following Bernoulli differential equation:*

$\sigma'(t)+a(t)\sigma(t) = b(t)\sigma(t)^n$. *With* $\sigma^2_{prior} \geq \sigma(0) > 0$, $n = 3$, $a(t) = -\frac{\gamma}{N}$, *and* $b(t) = -\frac{\gamma}{N}(NH_D(\mu_0)+\frac{1}{\sigma^2_{prior}})$. *This differential equation admit a solution and:*

$$\sigma(t) = \frac{\sigma_0 e^{\frac{\gamma t}{N}}}{\sqrt{1+N\sigma_0^2(H_D(\mu_0)+\frac{1}{N\sigma^2_{prior}})(e^{\frac{2\gamma t}{N}}-1)}} \tag{7}$$

Proof. First let's consider an interval $\mathcal{I}$, such that $\forall t \in \mathcal{I}, \sigma(t) > 0$. Such an interval exist because $\sigma(0) > 0$, and σ is continuous. Therefore on this interval we can define u, such that $u(t) = \frac{1}{\sigma(t)^2}$, and we have $u'(t) = -2\frac{\sigma'(t)}{\sigma(t)^3}$. Therefore by multiplying the differential equation by $\frac{-2}{\sigma(t)^3}$ we get:

$$u'(t) + \frac{2\gamma}{N}u(t) = \frac{2\gamma}{N}(NH_D(\mu_0) + \frac{1}{\sigma^2_{\text{prior}}}) \tag{8}$$

This is a first-order linear differential equation. Since $u(0) = \frac{1}{\sigma_0^2}$, the solution is :

$$u(t) = \frac{1}{\sigma_0^2}e^{-\frac{2\gamma}{N}t} + e^{-\frac{2\gamma}{N}t}\int_0^t \frac{2\gamma}{N}(NH_D(\mu_0)+\frac{1}{\sigma^2_{\text{prior}}})e^{\frac{2\gamma}{N}x}dx \tag{9}$$

By computing the integral, and inserting $\sigma(t)$ into the equation we obtain:

$$\frac{1}{\sigma(t)^2} = \frac{1}{\sigma_0^2}e^{-\frac{2\gamma}{N}t} + e^{-\frac{2\gamma}{N}t}(NH_D(\mu_0)+\frac{1}{\sigma^2_{\text{prior}}}(e^{\frac{2\gamma}{N}t}-1)) \tag{10}$$

$$\sigma(t)^2 = \frac{1}{\frac{1}{\sigma_0^2}e^{-\frac{2\gamma}{N}t} + e^{-\frac{2\gamma}{N}t}(NH_D(\mu_0)+\frac{1}{\sigma^2_{\text{prior}}}(e^{\frac{2\gamma}{N}t}-1))} \tag{11}$$

$$\sigma(t)^2 = \frac{\sigma_0^2 e^{\frac{2\gamma}{N}t}}{1+\sigma_0(NH_D(\mu_0)+\frac{1}{\sigma^2_{\text{prior}}}(e^{\frac{2\gamma}{N}t}-1))} \tag{12}$$

Finally, the element of the equation is passed to the square root. And knowing that the only viable solutions are when $\sigma(t) > 0$, we get the final results:

$$\boxed{\sigma(t) = \frac{\sigma_0 e^{\frac{\gamma t}{N}}}{\sqrt{1+N\sigma_0^2(H_D(\mu_0)+\frac{1}{N\sigma^2_{\text{prior}}})(e^{\frac{2\gamma t}{N}}-1)}}} \tag{13}$$

□

Proposition 2 (Study of a sequence's asymptotic behavior)**.** *Consider the sequence* $\alpha_{t+1} = \alpha_t(1-\alpha_t^2)$ *where* $0 < \alpha_0 < 1$. *Then, the sequence on* (α_t) *converge to zero, and :*

$$\lim_{t\to+\infty} \alpha_t\sqrt{2t} = 1. \tag{14}$$

Proof. (i): By induction, we establish that: $\forall t \in \mathbb{N}^*$, $\alpha_t \leq \frac{1}{\sqrt{2t}}$.

Base Case (Initialization): t=1,2

We define $f(x) = x(1-x^2)$. A simple analysis of the function gives f increasing over the interval $[0, \frac{1}{\sqrt{3}}]$ and decreasing over the interval $[\frac{1}{\sqrt{3}}, 1]$ reaching its maximum value of $\frac{2}{3\sqrt{3}}$ at $\frac{1}{\sqrt{3}}$.

Thus, $\alpha_1 \leq \frac{2}{3\sqrt{3}} \leq \frac{1}{\sqrt{2}}$ and $\alpha_2 \leq \frac{2}{3\sqrt{3}} \leq \frac{1}{\sqrt{4}}$, confirming the base case.

Inductive Step: We assume the proposition holds for $t \geq 2 \in \mathbb{N}$, i.e., $\alpha_t \leq \frac{1}{\sqrt{2t}}$.

As f is monotonically increasing on $[0, \frac{1}{\sqrt{3}}]$, and from the inductive hypothesis $f(\alpha_t) \leq f(\frac{1}{\sqrt{2t}})$ provided:

$$\alpha_{t+1} \leq \frac{1}{\sqrt{2t}}(1 - \frac{1}{2t}). \tag{15}$$

Using Taylor's expansion we have the following expression :

$$\frac{1}{\sqrt{2t+2}} = \frac{1}{\sqrt{2t}}(1 - \frac{1}{2t} + \frac{3}{8t^2} + R_3(\frac{1}{t})). \tag{16}$$

Where R_3 represents the remainder of the Taylor series and Taylor's inequality gives $\forall t \geq 2$, $|R_3(\frac{1}{t})| < \frac{3}{8t^2}$. Eq.(15) and Eq.(16) lead to $\alpha_{t+1} \leq \frac{1}{\sqrt{2t+2}}$

$$\forall t \in \mathbb{N}^*, \alpha_t \leq \frac{1}{\sqrt{2t}}. \tag{17}$$

(ii): We consider a sequence (V_t) defined as: $V_t = \frac{t}{2} - t^2\alpha_t^2$ and demonstrate that the sequence (V_t) is monotonically increasing. Taking the difference between successive terms, we have:

$$V_{t+1} - V_t = \frac{1}{2} - (t+1)^2\alpha_{t+1}^2 + t^2\alpha_t^2. \tag{18}$$

Using the result from (i) we ascertain that: $(t+1)^2\alpha_{t+1}^2 \leq \frac{1}{2}$.

From which we infer $V_{t+1} - V_t \geq t^2\alpha_t^2 \geq 0$

Involving that the sequence (Vt) is increasing. There are two possibilities: First possibility, (V_t) converges and the limit is:

$$\lim_{t\to+\infty} V_t = c \in \mathbb{R}. \tag{19}$$

Consequently:

$$\lim_{t\to+\infty} \frac{V_t}{t} = \lim_{t\to+\infty} t(\frac{1}{2t} - \alpha_t^2) = 0. \tag{20}$$

Concluding the proof when (V_t) converge:

$$\boxed{\lim_{t\to+\infty} \alpha_t\sqrt{2t} = 1}. \tag{21}$$

Second possibility:

$$\lim_{t\to+\infty} V_t = +\infty. \tag{22}$$

(iii): We consider the sequence (P_t) defined by: $P_t = \alpha_t\sqrt{2t}$.

We demonstrate that there is a given $t_0 \in \mathbb{N}^*$ such that $\forall t \geq t_0$, $P_t \leq P_{t+1}$. Involving (P_t) increasing beyond t_0.

Looking at the ratio between successive terms, we find:

$$\frac{P_{t+1}}{P_t} = \frac{\alpha_t(1-\alpha_t^2)\sqrt{2t+2}}{\alpha_t\sqrt{2t}} = (1-\alpha_t^2)\sqrt{1+\frac{1}{t}}. \tag{23}$$

Further expanding, we have:

$$\frac{P_{t+1}}{P_t} = 1 - \alpha_t^2 + \frac{1}{2t} + O(\frac{1}{t^2}). \tag{24}$$

Using the results from (i), we ascertain $\frac{1}{2t} - \alpha_t^2 \geq 0$. Additionally, by invoking (ii), it becomes clear that as t grows, the term $O(\frac{1}{t^2})$ become negligible relative to $(\frac{1}{2t} - \alpha_t^2)$. Consequently, we can deduce that there exists a particular $t_0 \in \mathbb{N}^*$ for which all $t \geq t_0$, $\frac{P_{t+1}}{P_t} \geq 1$.

Concluding to:

$$\exists t_0 \in \mathbb{N}^*, \forall t \geq t_0, (P_t) \nearrow. \tag{25}$$

From (i) we know that $(P_t) \leq 1$ and from (iii) we know that (P_t) is monotically increasing and so (P_t) converge:

$$\lim_{t\to+\infty} P_t = c. \tag{26}$$

(iv) We consider $(\log(P_t))$ demonstrating the convergence of the series $\sum(\frac{1}{2t} - \alpha_t^2)$.

First, let us rewrite (α_t).

$$\alpha_{t+2} = \alpha_{t+1}(1-\alpha_{t+1}^2) = \alpha_t(1-\alpha_t^2)(1-\alpha_{t+1}^2). \tag{27}$$

From the above recursion relation, we can inductively deduce the general form:

$$\alpha_t = \prod_{k=1}^{t-1}(1-\alpha_k^2)\alpha_1. \tag{28}$$

This allows us to express $(\log(P_t))$ as:

$$\log(P_t) = \sum_{k=1}^{t-1}\log((1-\alpha_k^2)) + \log(\alpha_1) + \log(\sqrt{2t}), \tag{29}$$

which, in the limit as n approaches infinity, is asymptotic to:

$$\log(P_t) \underset{t\to+\infty}{\sim} \sum_{k=1}^{n-1}\log((1-\alpha_k^2)) + \frac{1}{2}\log(t). \tag{30}$$

From a prior result (i), we have:

$$\log((1-\alpha_k^2)) = -\alpha_k^2 + O(\frac{1}{t^2}). \tag{31}$$

Invoking the properties of the Harmonic series, we can further deduce:

$$\log(P_t) \underset{t\to+\infty}{\sim} \sum_{k=1}^{n-1} -\alpha_k^2 + \frac{1}{2}\sum_{k=1}^{t-1}\frac{1}{k}. \tag{32}$$

Given Equation (26):

$$\lim_{t\to+\infty} \log(P_t) = \log(c). \tag{33}$$

In consequence the series $\sum(\frac{1}{2t} - \alpha_t^2)$ converge.

Based on previous results, namely (i) and (iii), we know that $(\frac{1}{2t} - \alpha_t^2) \geq 0$ and the sequence $(\frac{1}{2t} - \alpha_t^2)$ is monotonically decreasing.

We consider the two sequences (u_t) and (v_t) such that $u_t = \frac{1}{2t} - \alpha_t^2$ and $v_t = t(u_t - u_{t+1})$. By rearranging terms, the sum of the first t terms of v_k can be expressed as:

$$\sum_{k=0}^{t} v_k = \sum_{k=1}^{t} k u_k - \sum_{k=1}^{t+1}(k-1)u_k, \tag{34}$$

which gives:

$$\sum_{k=0}^{t} u_k = \sum_{k=0}^{t} v_k + t u_{t+1}. \tag{35}$$

Given that $\sum u_k$ converges in (iv), it follows that $(\sum_{k=0}^{t} v_k + t u_{t+1})$ converges too. Since $u_t \geq 0$ and the sequence (u_t) is monotonically decreasing, $v_t \geq 0$. Hence, both of the series $\sum v_k$ and $t u_{t+1}$ converge.

Considering the limit of the product $t u_t$, we have:

$$\lim_{t\to+\infty} t u_t = l. \tag{36}$$

If $l > 0$, we have $u_t \underset{t\to+\infty}{\sim} \frac{l}{t}$. However, this would lead to the divergence of $\sum u_k$ and consequently we deduce that $l = 0$ and therefore:

$$\lim_{t\to+\infty} t u_t = 0, \tag{37}$$

from which we can deduce:

$$\lim_{t\to+\infty} \frac{V_t}{t} = \lim_{t\to+\infty} t(\frac{1}{2t} - \alpha_t^2) = 0. \tag{38}$$

Concluding the proof when $V_t \to \infty$.:

$$\boxed{\lim_{t\to+\infty} \alpha_t\sqrt{2t} = 1} \tag{39}$$

□

Supplementary Note 4: Theorem demonstrations

Theorem 1 (MESU). *Consider a stream of data* $\{\mathcal{D}_i\}_{i=0}^t$. *Let* $q_{\boldsymbol{\theta}_t}(\boldsymbol{\omega})$ *be a mean-field Gaussian for a Bayesian neural network at time* t, *with* $\boldsymbol{\theta}_t = (\boldsymbol{\mu}_t, \boldsymbol{\sigma}_t)$ *and samples* $\boldsymbol{\omega} = \boldsymbol{\mu}_t + \boldsymbol{\epsilon} \cdot \boldsymbol{\sigma}_t$, $\boldsymbol{\epsilon} \sim \mathcal{N}(\vec{0}, \mathbf{I})$. *Suppose* $q_{\boldsymbol{\theta}_{t-1}}(\boldsymbol{\omega}) \approx p\big(\boldsymbol{\omega} \,|\, \mathcal{D}_{t-N-1}, \ldots, \mathcal{D}_{t-1}\big)$, *and that each dataset* $\mathcal{D}_i$ *has equal marginal likelihood. Under a second-order expansion of* $\mathcal{C}_t$ *around* $\boldsymbol{\mu}_{t-1}$ *and* $\boldsymbol{\sigma}_{t-1}$, *plus the small-update assumption* $|\frac{\Delta\sigma_i}{\sigma_{i,t-1}}| \ll 1$, *the parameter updates become:*

$$\Delta\boldsymbol{\sigma} = -\frac{\boldsymbol{\sigma}_{t-1}^2}{2}\frac{\partial \mathcal{C}_t}{\partial \boldsymbol{\sigma}_{t-1}} + \frac{\boldsymbol{\sigma}_{t-1}}{2\,N\,\boldsymbol{\sigma}_{prior}^2}\big(\boldsymbol{\sigma}_{prior}^2 - \boldsymbol{\sigma}_{t-1}^2\big), \tag{40}$$

$$\Delta\boldsymbol{\mu} = -\boldsymbol{\sigma}_{t-1}^2\frac{\partial \mathcal{C}_t}{\partial \boldsymbol{\mu}_{t-1}} + \frac{\boldsymbol{\sigma}_{t-1}^2}{N\,\boldsymbol{\sigma}_{prior}^2}\big(\boldsymbol{\mu}_{prior} - \boldsymbol{\mu}_{t-1}\big). \tag{41}$$

Proof. In the main article, we have seen that the assumptions stated in the theorem lead us to the following free variational energy to minimize:

$$\mathcal{F}_t = \underbrace{\big[D_{KL}[q_{\boldsymbol{\theta}_t}(\boldsymbol{\omega})||q_{\boldsymbol{\theta}_{t-1}}(\boldsymbol{\omega})] + \mathcal{C}_t\big]}_{\text{Bayesian continual learning}} + \underbrace{\frac{1}{N}\left[-\frac{(\boldsymbol{\mu}_t - \boldsymbol{\mu}_{\mathrm{L}_{t-1}})^2}{2\boldsymbol{\sigma}_{\mathrm{L}_{t-1}}^2} - \frac{\boldsymbol{\sigma}_t^2}{\boldsymbol{\sigma}_{\mathrm{L}_{t-1}}^2}\right]}_{\text{forgetting}}. \tag{42}$$

Therefore, to find the optimal update over sigma, we have to find the solution to the following equation:

$$\frac{\partial \mathcal{F}_t}{\partial \boldsymbol{\sigma}_t} = -\frac{1}{\boldsymbol{\sigma}_{t-1} + \Delta\boldsymbol{\sigma}} + \frac{\boldsymbol{\sigma}_{t-1} + \Delta\boldsymbol{\sigma}}{\boldsymbol{\sigma}_{t-1}^2} + \frac{\partial \mathcal{C}_t}{\partial \boldsymbol{\sigma}_t} - \frac{2(\boldsymbol{\sigma}_{t-1} + \Delta\boldsymbol{\sigma})}{N\boldsymbol{\sigma}_{L_{t-1}}^2} = 0. \tag{43}$$

In second order approximation $\frac{\partial \mathcal{C}_t}{\partial \boldsymbol{\sigma}_t} = \frac{\partial \mathcal{C}_t}{\partial \boldsymbol{\sigma}_{t-1}} + \Delta\boldsymbol{\sigma}\frac{\partial^2 \mathcal{C}_t}{\partial \boldsymbol{\sigma}_{t-1}^2}$. Therefore according to Lemma. 1 and Lemma. 2, $\frac{\partial \mathcal{C}_t}{\partial \boldsymbol{\sigma}_t} \approx \frac{\partial \mathcal{C}_t}{\partial \boldsymbol{\sigma}_{t-1}}(1 + \frac{\Delta\boldsymbol{\sigma}}{\sigma})$. So if we assume $|\frac{\Delta\sigma_i}{\sigma_{i,t-1}}| \ll 1$, $\frac{\partial \mathcal{C}_t}{\partial \boldsymbol{\sigma}_t} \approx \frac{\partial \mathcal{C}_t}{\partial \boldsymbol{\sigma}_{t-1}}$, and the equation is no longer implicit. We obtain:

$$\frac{\partial \mathcal{F}_t}{\partial \boldsymbol{\sigma}_t} = -\frac{1}{\boldsymbol{\sigma}_{t-1} + \Delta\boldsymbol{\sigma}} + \frac{\boldsymbol{\sigma}_{t-1} + \Delta\boldsymbol{\sigma}}{\boldsymbol{\sigma}_{t-1}^2} + \frac{\partial \mathcal{C}_t}{\partial \boldsymbol{\sigma}_{t-1}} - \frac{2(\boldsymbol{\sigma}_{t-1} + \Delta\boldsymbol{\sigma})}{N\boldsymbol{\sigma}_{L_{t-1}}^2} = 0 \tag{44}$$

Which gives:

$$-\frac{\boldsymbol{\sigma}_{t-1}^2}{\boldsymbol{\sigma}_{t-1}(1 + \frac{\Delta\boldsymbol{\sigma}}{\boldsymbol{\sigma}_{t-1}})} + (\boldsymbol{\sigma}_{t-1} + \Delta\boldsymbol{\sigma}) = -\boldsymbol{\sigma}_{t-1}^2\frac{\partial \mathcal{C}_t}{\partial \boldsymbol{\sigma}_{t-1}} + \frac{2\boldsymbol{\sigma}_{t-1}^2(\boldsymbol{\sigma}_{t-1} + \Delta\boldsymbol{\sigma})}{N\boldsymbol{\sigma}_{L_{t-1}}^2} \tag{45}$$

Since $|\frac{\Delta\boldsymbol{\sigma}}{\boldsymbol{\sigma}_{t-1}}| \ll 1$, we obtain:

$$2\Delta\boldsymbol{\sigma}\left(1 - \frac{\boldsymbol{\sigma}_{t-1}^2}{N\boldsymbol{\sigma}_{L_{t-1}}^2}\right) = -\boldsymbol{\sigma}_{t-1}^2\frac{\partial \mathcal{C}_t}{\partial \boldsymbol{\sigma}_{t-1}} + \frac{2\boldsymbol{\sigma}_{t-1}^3}{N\boldsymbol{\sigma}_{L_{t-1}}^2} \tag{46}$$

We will always choose $N \gg 1$, and since we have:

$$\frac{1}{\boldsymbol{\sigma}_{t-1}^2} = \frac{1}{\boldsymbol{\sigma}_{L_{t-1}}^2} + \frac{1}{\boldsymbol{\sigma}_{\text{prior}}^2} \tag{47}$$

Therefore, we obtain $|\frac{\boldsymbol{\sigma}_{t-1}^2}{N\boldsymbol{\sigma}_{L_{t-1}}^2}| \ll 1$ and:

$$\Delta\boldsymbol{\sigma} = -\frac{\boldsymbol{\sigma}_{t-1}^2}{2}\frac{\partial \mathcal{C}_t}{\partial \boldsymbol{\sigma}_{t-1}} + \frac{\boldsymbol{\sigma}_{t-1}^3}{N}\left(\frac{1}{\boldsymbol{\sigma}_{t-1}^2} - \frac{1}{\boldsymbol{\sigma}_{\text{prior}}^2}\right) \tag{48}$$

Which can be rewritten:

$$\boxed{\Delta\boldsymbol{\sigma} = -\frac{\boldsymbol{\sigma}_{t-1}^2}{2}\frac{\partial \mathcal{C}_t}{\partial \boldsymbol{\sigma}_{t-1}} + \frac{\boldsymbol{\sigma}_{t-1}}{2N\boldsymbol{\sigma}_{\text{prior}}^2}(\boldsymbol{\sigma}_{\text{prior}}^2 - \boldsymbol{\sigma}_{t-1}^2)} \tag{49}$$

In the main article, we have seen that the assumptions stated in the theorem lead us to the following free variational energy to minimize:

$$\mathcal{F}_t = \underbrace{\left[D_{KL}[q_{\boldsymbol{\theta}_t}(\boldsymbol{\omega})||q_{\boldsymbol{\theta}_{t-1}}(\boldsymbol{\omega})] + \mathcal{C}_t\right]}_{\text{Bayesian continual learning}} + \underbrace{\frac{1}{N}\left[-\frac{(\boldsymbol{\mu}_t - \boldsymbol{\mu}_{\text{L}_{t-1}})^2}{2\boldsymbol{\sigma}_{\text{L}_{t-1}}^2} - \frac{\boldsymbol{\sigma}_t^2}{\boldsymbol{\sigma}_{\text{L}_{t-1}}^2}\right]}_{\text{forgetting}}. \tag{50}$$

Therefore, to find the optimal update over mu, we have to find the solution to the following equation:

$$\frac{\partial \mathcal{F}_t}{\partial \boldsymbol{\mu}_t} = \frac{\Delta\boldsymbol{\mu}}{\boldsymbol{\sigma}_{t-1}^2} + \frac{\partial \mathcal{C}_t}{\partial \boldsymbol{\mu}_t} - \frac{\boldsymbol{\mu}_t - \boldsymbol{\mu}_{\text{L}}}{N\boldsymbol{\sigma}_{L_{t-1}}^2} = 0, \tag{51}$$

In second order approximation $\frac{\partial \mathcal{C}_t}{\partial \boldsymbol{\mu}_t} = \frac{\partial \mathcal{C}_t}{\partial \boldsymbol{\mu}_{t-1}} + \Delta\boldsymbol{\mu}\frac{\partial^2 \mathcal{C}_t}{\partial \boldsymbol{\mu}_{t-1}^2}$, and the equation is no longer implicit. We obtain:

$$\frac{\partial \mathcal{F}_t}{\partial \boldsymbol{\mu}_t} = \frac{\Delta\boldsymbol{\mu}}{\boldsymbol{\sigma}_{t-1}^2} + \frac{\partial \mathcal{C}_t}{\partial \boldsymbol{\mu}_{t-1}} + \Delta\boldsymbol{\mu}\frac{\partial^2 \mathcal{C}_t}{\partial \boldsymbol{\mu}_{t-1}^2} - \frac{\boldsymbol{\mu}_t - \boldsymbol{\mu}_{\text{L}}}{N\boldsymbol{\sigma}_{L_{t-1}}^2} = 0 \tag{52}$$

Which can be re-arranged as :

$$\Delta\boldsymbol{\mu}(1 + \boldsymbol{\sigma}_{t-1}^2\frac{\partial^2 \mathcal{C}_t}{\partial \boldsymbol{\mu}_{t-1}^2}) = -\boldsymbol{\sigma}_{t-1}^2\frac{\partial \mathcal{C}_t}{\partial \boldsymbol{\mu}_{t-1}} + \frac{\boldsymbol{\sigma}_{t-1}^2}{N\boldsymbol{\sigma}_{L_{t-1}}^2}(\boldsymbol{\mu}_t - \boldsymbol{\mu}_{\text{L}}) \tag{53}$$

According to Lemma. 2, $\boldsymbol{\sigma}_{t-1}^2\frac{\partial^2 \mathcal{C}_t}{\partial \boldsymbol{\mu}_{t-1}^2} = \boldsymbol{\sigma}_{t-1}\frac{\partial \mathcal{C}_t}{\partial \boldsymbol{\sigma}_{t-1}}$, and since $|\frac{\Delta\boldsymbol{\sigma}}{\boldsymbol{\sigma}_{t-1}}| \ll 1$, and $N \gg 1$, we have $|\boldsymbol{\sigma}_{t-1}\frac{\partial^2 \mathcal{C}_t}{\partial \boldsymbol{\sigma}_{t-1}^2}| \ll 1$. Therefore, we can simplify to:

$$\Delta\boldsymbol{\mu} = -\boldsymbol{\sigma}_{t-1}^2\frac{\partial \mathcal{C}_t}{\partial \boldsymbol{\mu}_{t-1}} + \frac{\boldsymbol{\sigma}_{t-1}^2}{N\boldsymbol{\sigma}_{L_{t-1}}^2}(\boldsymbol{\mu}_t - \frac{(\boldsymbol{\sigma}_{L_{t-1}}^2 + \boldsymbol{\sigma}_{\text{prior}}^2)\boldsymbol{\mu}_{t-1} - \boldsymbol{\mu}_{\text{prior}}\boldsymbol{\sigma}_{L_{t-1}}^2}{\boldsymbol{\sigma}_{\text{prior}}^2}) \tag{54}$$

$$\Delta\boldsymbol{\mu} = -\boldsymbol{\sigma}_{t-1}^2\frac{\partial \mathcal{C}_t}{\partial \boldsymbol{\mu}_{t-1}} + \frac{\boldsymbol{\sigma}_{t-1}^2}{N\boldsymbol{\sigma}_{L_{t-1}}^2}(\Delta\boldsymbol{\mu} - \frac{\boldsymbol{\sigma}_{L_{t-1}}^2}{\boldsymbol{\sigma}_{\text{prior}}^2}\boldsymbol{\mu}_{t-1} + \frac{N\boldsymbol{\sigma}_{L_{t-1}}^2}{\boldsymbol{\sigma}_{\text{prior}}^2}\boldsymbol{\mu}_{\text{prior}}) \tag{55}$$

$$\Delta\boldsymbol{\mu}(1 - \frac{\boldsymbol{\sigma}_{t-1}^2}{N\boldsymbol{\sigma}_{L_{t-1}}^2}) = -\boldsymbol{\sigma}_{t-1}^2\frac{\partial \mathcal{C}_t}{\partial \boldsymbol{\mu}_{t-1}} - \frac{\boldsymbol{\sigma}_{t-1}^2}{N\boldsymbol{\sigma}_{\text{prior}}^2}(\boldsymbol{\mu}_{t-1} - \boldsymbol{\mu}_{\text{prior}}) \tag{56}$$

We will always choose $N \gg 1$, and since we have:

$$\frac{1}{\boldsymbol{\sigma}_{t-1}^2} = \frac{1}{\boldsymbol{\sigma}_{L_{t-1}}^2} + \frac{1}{\boldsymbol{\sigma}_{\text{prior}}^2} \tag{57}$$

Therefore, we obtain $|\frac{\boldsymbol{\sigma}_{t-1}^2}{N\boldsymbol{\sigma}_{L_{t-1}}^2}| \ll 1$ and:

$$\boxed{\Delta\boldsymbol{\mu} = -\boldsymbol{\sigma}_{t-1}^2 \frac{\partial \mathcal{C}_t}{\partial \boldsymbol{\mu}_{t-1}} + \frac{\boldsymbol{\sigma}_{t-1}^2}{N\boldsymbol{\sigma}_{\text{prior}}^2}(\boldsymbol{\mu}_{\text{prior}} - \boldsymbol{\mu}_{t-1})} \tag{58}$$

□

Theorem 2 (Newton's method in variational inference)**.** *Let* $q_{\boldsymbol{\theta}}(\boldsymbol{\omega}) = \mathcal{N}\big(\boldsymbol{\omega}; \boldsymbol{\mu}, \text{diag}(\boldsymbol{\sigma}^2)\big)$ *be a mean-field Gaussian for a Bayesian neural network, with* $\boldsymbol{\omega} = \boldsymbol{\mu} + \boldsymbol{\epsilon} \cdot \boldsymbol{\sigma}$*,* $\boldsymbol{\epsilon} \sim \mathcal{N}(\vec{0}, \mathbf{I}_s)$*, and with a prior* $p(\boldsymbol{\omega}) = \mathcal{N}\big(\boldsymbol{\omega}; \boldsymbol{\mu}_{prior}, \text{diag}(\boldsymbol{\sigma}_{prior}^2)\big)$*. Given* $\mathcal{D}$ *split into* N *i.i.d. mini-batches, and defining* $\mathcal{C} = \mathbb{E}_{\boldsymbol{\epsilon}}[\mathcal{L}(\boldsymbol{\omega})]$*, a diagonal Newton update for* $\boldsymbol{\sigma}$ *and* $\boldsymbol{\mu}$ *with learning rate* γ *and* $\boldsymbol{\sigma} \approx \boldsymbol{\sigma}_{post}$ *becomes:*

$$\Delta\boldsymbol{\sigma} = \frac{\gamma N}{2}\Big[t - \boldsymbol{\sigma}^2 \frac{\partial \mathcal{C}}{\partial \boldsymbol{\sigma}} + \frac{\boldsymbol{\sigma}}{N\,\boldsymbol{\sigma}_{prior}^2}\big(\boldsymbol{\sigma}_{prior}^2 - \boldsymbol{\sigma}^2\big)\Big], \tag{59}$$

$$\Delta\boldsymbol{\mu} = \gamma N\Big[-\boldsymbol{\sigma}^2 \frac{\partial \mathcal{C}}{\partial \boldsymbol{\mu}} + \frac{\boldsymbol{\sigma}^2}{N\,\boldsymbol{\sigma}_{prior}^2}\big(\boldsymbol{\mu}_{prior} - \boldsymbol{\mu}\big)\Big]. \tag{60}$$

Proof. In the main article, we have seen that the assumptions stated in the theorem lead us to the following free variational energy to minimize:

$$\mathcal{F} = D_{KL}[q_\theta(\boldsymbol{\omega})||p(\boldsymbol{\omega}|D)] = \log\frac{\boldsymbol{\sigma}_{\text{post}}}{\boldsymbol{\sigma}} + \frac{\boldsymbol{\sigma}^2 + (\boldsymbol{\mu} - \boldsymbol{\mu}_{\text{post}})^2}{2\boldsymbol{\sigma}_{\text{post}}^2} - \frac{1}{2},$$

with:

$$\frac{1}{\boldsymbol{\sigma}_{\text{post}}^2} = \frac{1}{\boldsymbol{\sigma}_{\text{L}}^2} + \frac{1}{\boldsymbol{\sigma}_{\text{prior}}^2}, \tag{61}$$

From those equations, we can obtain an expression of the first and second-order derivative of the free variational energy concerning $\boldsymbol{\sigma}$.

$$\frac{\partial \mathcal{F}}{\partial \boldsymbol{\sigma}} = -\frac{1}{\boldsymbol{\sigma}} + \boldsymbol{\sigma}(\frac{1}{\boldsymbol{\sigma}_{\text{L}}^2} + \frac{1}{\boldsymbol{\sigma}_{\text{prior}}^2}). \tag{62}$$

$$\frac{\partial^2 \mathcal{F}}{\partial \boldsymbol{\sigma}^2} = \frac{1}{\boldsymbol{\sigma}^2} + \frac{1}{\boldsymbol{\sigma}_{\text{L}}^2} + \frac{1}{\boldsymbol{\sigma}_{\text{prior}}^2}. \tag{63}$$

Therefore we can apply a diagonal approximation of Newton's method to minimize it:

$$\Delta\boldsymbol{\sigma} = -\gamma\frac{1}{\frac{\partial^2 \mathcal{F}}{\partial \boldsymbol{\sigma}^2}}\frac{\partial \mathcal{F}}{\partial \boldsymbol{\sigma}}. \tag{64}$$

Replacing the right end term in this equation we get:

$$\Delta \boldsymbol{\sigma} = -\gamma \frac{-\frac{1}{\boldsymbol{\sigma}} + \boldsymbol{\sigma}(\frac{1}{\boldsymbol{\sigma}_{\mathrm{L}}^2} + \frac{1}{\boldsymbol{\sigma}_{\mathrm{prior}}^2})}{\frac{1}{\boldsymbol{\sigma}^2} + \frac{1}{\boldsymbol{\sigma}_{\mathrm{L}}^2} + \frac{1}{\boldsymbol{\sigma}_{\mathrm{prior}}^2}}. \tag{65}$$

We now multiply by $\boldsymbol{\sigma}^2$ the two terms of the fraction:

$$\Delta \boldsymbol{\sigma} = -\gamma \frac{-\boldsymbol{\sigma} + \boldsymbol{\sigma}(\frac{\boldsymbol{\sigma}^2}{\boldsymbol{\sigma}_{\mathrm{L}}^2} + \frac{\boldsymbol{\sigma}^2}{\boldsymbol{\sigma}_{\mathrm{prior}}^2})}{1 + \frac{\boldsymbol{\sigma}^2}{\boldsymbol{\sigma}_{\mathrm{L}}^2} + \frac{\boldsymbol{\sigma}^2}{\boldsymbol{\sigma}_{\mathrm{prior}}^2}}. \tag{66}$$

And rearranging the numerator we get:

$$\Delta \boldsymbol{\sigma} = -\gamma \frac{\frac{\boldsymbol{\sigma}^3}{\boldsymbol{\sigma}_{\mathrm{L}}^2} + \frac{\boldsymbol{\sigma}}{\boldsymbol{\sigma}_{\mathrm{prior}}^2}(\boldsymbol{\sigma}^2 - \boldsymbol{\sigma}_{\mathrm{prior}}^2)}{1 + \frac{\boldsymbol{\sigma}^2}{\boldsymbol{\sigma}_{\mathrm{L}}^2} + \frac{\boldsymbol{\sigma}^2}{\boldsymbol{\sigma}_{\mathrm{prior}}^2}}. \tag{67}$$

In the i.i.d scenario, according to Lemma. 1 and Lemma. 2, we have $\frac{1}{\boldsymbol{\sigma}_{\mathrm{L}}^2} = \frac{N}{\boldsymbol{\sigma}} \frac{\partial \mathcal{C}}{\partial \boldsymbol{\sigma}} = N H_D(\boldsymbol{\mu})$. This leads to:

$$\Delta \boldsymbol{\sigma} = -\gamma \frac{N\boldsymbol{\sigma}^2 \frac{\partial \mathcal{C}}{\partial \boldsymbol{\sigma}} + \frac{\boldsymbol{\sigma}}{\boldsymbol{\sigma}_{\mathrm{prior}}^2}(\boldsymbol{\sigma}^2 - \boldsymbol{\sigma}_{\mathrm{prior}}^2)}{1 + \frac{\boldsymbol{\sigma}^2}{\boldsymbol{\sigma}_{\mathrm{L}}^2} + \frac{\boldsymbol{\sigma}^2}{\boldsymbol{\sigma}_{\mathrm{prior}}^2}}. \tag{68}$$

Although the same approximation of $\frac{1}{\boldsymbol{\sigma}_{\mathrm{L}}^2}$ could be applied for the denominator, relying on a stochastic evaluation of the second order derivative in the denominator might be unreliable, as on a mini-batch $\frac{\partial \mathcal{C}}{\partial \boldsymbol{\sigma}}$ might even be negative. Therefore, another more stable approximation for the denominator can be taken if one takes $\boldsymbol{\sigma}$ to be $\boldsymbol{\sigma}_{\mathrm{post}}$, which will be confirmed when sigma has converged. This leads us to the following equation:

$$\frac{1}{\boldsymbol{\sigma}^2} = \frac{1}{\boldsymbol{\sigma}_{\mathrm{post}}^2} = \frac{1}{\boldsymbol{\sigma}_{\mathrm{L}}^2} + \frac{1}{\boldsymbol{\sigma}_{\mathrm{prior}}^2}, \tag{69}$$

Therefore, when we are close enough to convergence :

$$1 + \frac{\boldsymbol{\sigma}^2}{\boldsymbol{\sigma}_{\mathrm{L}}^2} + \frac{\boldsymbol{\sigma}^2}{\boldsymbol{\sigma}_{\mathrm{prior}}^2} \approx 2, \tag{70}$$

And we arrive to the final update:

$$\boxed{\Delta \boldsymbol{\sigma} = \frac{\gamma N}{2} \left(-\boldsymbol{\sigma}^2 \frac{\partial \mathcal{C}}{\partial \boldsymbol{\sigma}} + \frac{\boldsymbol{\sigma}}{N \boldsymbol{\sigma}_{\mathrm{prior}}^2} \left(\boldsymbol{\sigma}_{\mathrm{prior}}^2 - \boldsymbol{\sigma}^2\right) \right)}. \tag{71}$$

In the main article, we have seen that the assumptions stated in the theorem lead us to the following free variational energy to minimize:

$$\mathcal{F} = D_{KL}[q_\theta(\boldsymbol{\omega})||p(\boldsymbol{\omega}|D)] = \log \frac{\boldsymbol{\sigma}_{\mathrm{post}}}{\boldsymbol{\sigma}} + \frac{\boldsymbol{\sigma}^2 + (\boldsymbol{\mu} - \boldsymbol{\mu}_{\mathrm{post}})^2}{2\boldsymbol{\sigma}_{\mathrm{post}}^2} - \frac{1}{2},$$

with:

$$\frac{1}{\boldsymbol{\sigma}_{\text{post}}^2} = \frac{1}{\boldsymbol{\sigma}_{\text{L}}^2} + \frac{1}{\boldsymbol{\sigma}_{\text{prior}}^2}, \tag{72}$$

$$\boldsymbol{\mu}_{\text{post}} = \frac{\boldsymbol{\mu}_{\text{L}}\boldsymbol{\sigma}_{\text{prior}}^2 + \boldsymbol{\mu}_{\text{prior}}\boldsymbol{\sigma}_{\text{L}}^2}{\boldsymbol{\sigma}_{\text{L}}^2 + \boldsymbol{\sigma}_{\text{prior}}^2}. \tag{73}$$

From those equations, we can obtain an expression of the first and second-order derivative of the free variational energy concerning $\boldsymbol{\mu}$.

$$\frac{\partial \mathcal{F}}{\partial \boldsymbol{\mu}} = \frac{\boldsymbol{\mu} - \boldsymbol{\mu}_{\text{post}}}{\boldsymbol{\sigma}_{\text{post}}^2} \tag{74}$$

$$\frac{\partial^2 \mathcal{F}}{\partial \boldsymbol{\mu}^2} = \frac{1}{\boldsymbol{\sigma}_{\text{post}}^2} = \frac{1}{\boldsymbol{\sigma}_{\text{L}}^2} + \frac{1}{\boldsymbol{\sigma}_{\text{prior}}^2}. \tag{75}$$

Therefore we can apply a diagonal approximation of Newton's method to minimize it:

$$\Delta\boldsymbol{\mu} = -\gamma \frac{1}{\frac{\partial^2 \mathcal{F}}{\partial \boldsymbol{\mu}^2}} \frac{\partial \mathcal{F}}{\partial \boldsymbol{\mu}}. \tag{76}$$

Without forgetting that classically the free variational energy can be expressed as :

$$\mathcal{F} = D_{KL}[q_{\boldsymbol{\theta}}(\boldsymbol{\omega})||p(\boldsymbol{\omega})] - \mathbb{E}_{q_{\boldsymbol{\theta}}(\boldsymbol{\omega})} \log p(\mathcal{D}|\boldsymbol{\omega}). \tag{77}$$

With a Gaussian prior, and our notation, the derivative of the free variational energy can be expressed as :

$$\frac{\partial \mathcal{F}}{\partial \boldsymbol{\mu}} = N\frac{\partial \mathcal{C}}{\partial \boldsymbol{\mu}} + \frac{\boldsymbol{\mu} - \boldsymbol{\mu}_{\text{prior}}}{\boldsymbol{\sigma}_{\text{prior}}^2} \tag{78}$$

Replacing the right end term in this equation we get:

$$\Delta\boldsymbol{\mu} = -\gamma \frac{N\frac{\partial \mathcal{C}}{\partial \boldsymbol{\mu}} + \frac{\boldsymbol{\mu} - \boldsymbol{\mu}_{\text{prior}}}{\boldsymbol{\sigma}_{\text{prior}}^2}}{\frac{1}{\boldsymbol{\sigma}_{\text{L}}^2} + \frac{1}{\boldsymbol{\sigma}_{\text{prior}}^2}}. \tag{79}$$

We now multiply by $\boldsymbol{\sigma}^2$ the two terms of the fraction:

$$\Delta\boldsymbol{\sigma} = -\gamma \frac{\boldsymbol{\sigma}^2 N\frac{\partial \mathcal{C}}{\partial \boldsymbol{\mu}} + \frac{\boldsymbol{\sigma}^2}{\boldsymbol{\sigma}_{\text{prior}}^2}(\boldsymbol{\mu} - \boldsymbol{\mu}_{\text{prior}})}{\frac{\boldsymbol{\sigma}^2}{\boldsymbol{\sigma}_{\text{L}}^2} + \frac{\boldsymbol{\sigma}^2}{\boldsymbol{\sigma}_{\text{prior}}^2}}. \tag{80}$$

In the i.i.d scenario, according to Lemma. 1 and Lemma. 2, we have $\frac{1}{\boldsymbol{\sigma}_{\text{L}}^2} = \frac{N}{\boldsymbol{\sigma}}\frac{\partial \mathcal{C}}{\partial \boldsymbol{\sigma}} = NH_D(\boldsymbol{\mu})$. We could approximate $\frac{1}{\boldsymbol{\sigma}_{\text{L}}^2}$. However relying on a stochastic evaluation of the second order derivative in the denominator might be unreliable, as on a mini-batch $\frac{\partial \mathcal{C}}{\partial \boldsymbol{\sigma}}$ might even be negative. Therefore, another more stable approximation for the denominator can be taken if one takes $\boldsymbol{\sigma}$ to be $\boldsymbol{\sigma}_{\text{post}}$, which will be confirmed when sigma has converged. This leads us to the following equation:

$$\frac{1}{\boldsymbol{\sigma}^2} = \frac{1}{\boldsymbol{\sigma}^2_{\text{post}}} = \frac{1}{\boldsymbol{\sigma}^2_{\text{L}}} + \frac{1}{\boldsymbol{\sigma}^2_{\text{prior}}}, \tag{81}$$

Therefore, when we are close enough to convergence :

$$\frac{\boldsymbol{\sigma}^2}{\boldsymbol{\sigma}^2_{\text{L}}} + \frac{\boldsymbol{\sigma}^2}{\boldsymbol{\sigma}^2_{\text{prior}}} \approx 1, \tag{82}$$

And we arrive to the final update:

$$\boxed{\Delta\boldsymbol{\mu} = \gamma N \left(-\boldsymbol{\sigma}^2 \frac{\partial \mathcal{C}}{\partial \boldsymbol{\mu}} + \frac{\boldsymbol{\sigma}^2}{N \boldsymbol{\sigma}^2_{\text{prior}}} (\boldsymbol{\mu}_{\text{prior}} - \boldsymbol{\mu}) \right)}. \tag{83}$$

□

Supplementary Note 5: Algorithm

Algorithm 1: MEtaplasticity from Synaptic Uncertainty

Let E the number of epochs to perform
Let B the number of batches to perform
Let S the number of samples for Monte Carlo integration
Let $p(\boldsymbol{\omega}) \sim (\boldsymbol{\mu}_{\text{prior}}, \boldsymbol{\sigma}^2_{\text{prior}})$ the prior distribution of the weights
for E **do**
  for B **do**
    for i *in* S **do**
      Sample $\boldsymbol{\epsilon}_i \sim \mathcal{N}(0, \boldsymbol{I}_d)$ with $\boldsymbol{I}_d$ the identity matrix of size d synapses.
      Compute the reparameterization trick for each weight $\boldsymbol{\omega}_i = \boldsymbol{\mu}_{t-1} + \boldsymbol{\sigma}_{t-1} \times \boldsymbol{\epsilon}_i$.
      Compute the loss function $\mathcal{L}(\boldsymbol{\omega}_i)$ with respect to weights $\boldsymbol{\omega}_i$.
      Compute the gradient of the loss $\frac{\partial \mathcal{L}(\boldsymbol{\omega}_i)}{\partial \boldsymbol{\omega}_i}$.
    end
    Compute the expectation of the gradients through Monte Carlo integration.

$$\frac{\partial \mathcal{C}_t}{\partial \boldsymbol{\mu}_{t-1}} \leftarrow \frac{1}{S} \sum_{i=0}^{S} \left[\frac{\partial \mathcal{L}(\boldsymbol{\omega}_i)}{\partial \boldsymbol{\omega}_i} \right],$$

$$\frac{\partial \mathcal{C}_t}{\partial \boldsymbol{\sigma}_{t-1}} \leftarrow \frac{1}{S} \sum_{i=0}^{S} \left[\frac{\partial \mathcal{L}(\boldsymbol{\omega}_i)}{\partial \boldsymbol{\omega}_i} \times \boldsymbol{\epsilon}_i \right].$$

    Update $\boldsymbol{\mu}$ and $\boldsymbol{\sigma}$.

$$\Delta \boldsymbol{\sigma} = -\frac{\boldsymbol{\sigma}^2_{t-1}}{2} \frac{\partial \mathcal{C}_t}{\partial \boldsymbol{\sigma}_{t-1}} + \frac{\boldsymbol{\sigma}_{t-1}}{2N\boldsymbol{\sigma}^2_{\text{prior}}} (\boldsymbol{\sigma}^2_{\text{prior}} - \boldsymbol{\sigma}^2_{t-1}),$$

$$\Delta \boldsymbol{\mu} = -\boldsymbol{\sigma}^2_{t-1} \frac{\partial \mathcal{C}_t}{\partial \boldsymbol{\mu}_{t-1}} + \frac{\boldsymbol{\sigma}^2_{t-1}}{N\boldsymbol{\sigma}^2_{\text{prior}}} (\boldsymbol{\mu}_{\text{prior}} - \boldsymbol{\mu}_{t-1}).$$

  end
end

Supplementary Note 6: Impact of the memory window N on the Permuted MNIST dataset

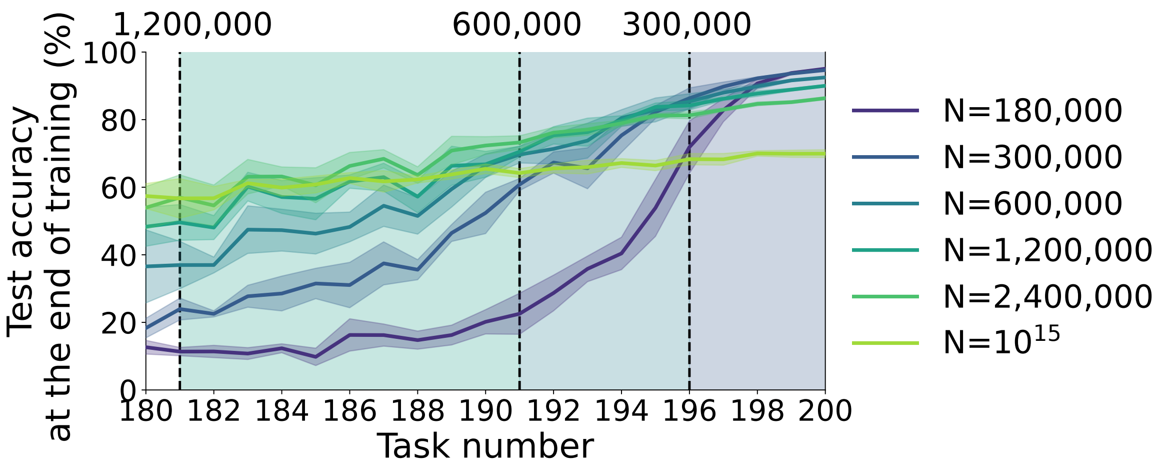

Supplementary Figure 2. Effect of the memory window N on accuracy. Test accuracy on the 200-task Permuted MNIST benchmark, presented for the last 20 tasks, and different memory windows. Vertical bars indicate the memory windows corresponding to N values of $300,000$, $600,000$ and $1,200,000$, as guides for the eyes. The unbounded case $N = 10^{15}$ behaves like FOO-VB Diagonal. Shaded areas around the curves show one standard deviation over five runs.

This Note investigates how the memory window N, the number of training iterations whose information is retained by MESU, affects performance on the 200-task Permuted MNIST benchmark, using the same hyper-parameters as in the main text.

Supplementary Fig. 2 compares six values of N: 180,000, 300,000, 600,000, 1,200,000, 2,400,000, and 10^{15}. Given that each Permuted MNIST task contains $60,000$ training samples, these values correspond approximately to memory windows of 3, 5, 10, 20, 40 tasks, and an effectively unbounded window (the latter reproduces the behavior of FOO-VB Diagonal, which has no forgetting mechanism). It is presented with 50 hidden units (the network used in Fig. 3 of the main article).

In this particular network, to maximize accuracy over the last five tasks (tasks 196 to 200), the optimal N value is 300,000 (yielding a five-last-tasks mean value 91.3%), which corresponds to a memory window of five tasks. A lower N value suffers from too much catastrophic forgetting (e.g., with a N value of 180,000 the five-last-tasks mean accuracy is 86.9%). Conversely higher N values suffer from catastrophic remembering: large N values protect older knowledge at the expense of plasticity, lowering short-term (last five tasks) accuracy (e.g., with $N = 2,400,000$, the five-last-tasks mean accuracy is 84.1%, and with $N = 10^{15}$ only 69.3%). A hypothesis for explaining this behavior is that the neural network has a limited capacity in how many tasks it can reliably remember, and therefore accuracy is degraded when trying to remember more than it can accommodate.

To verify this intuition that the optimal N value is network-capacity limited, we repeated the experiment with larger networks containing 256, 512, and 1024 hidden units (Supplementary Fig. 3). For each size, we evaluated the same five finite windows and the $N = 10^{15}$ case. Supplementary Fig. 3a

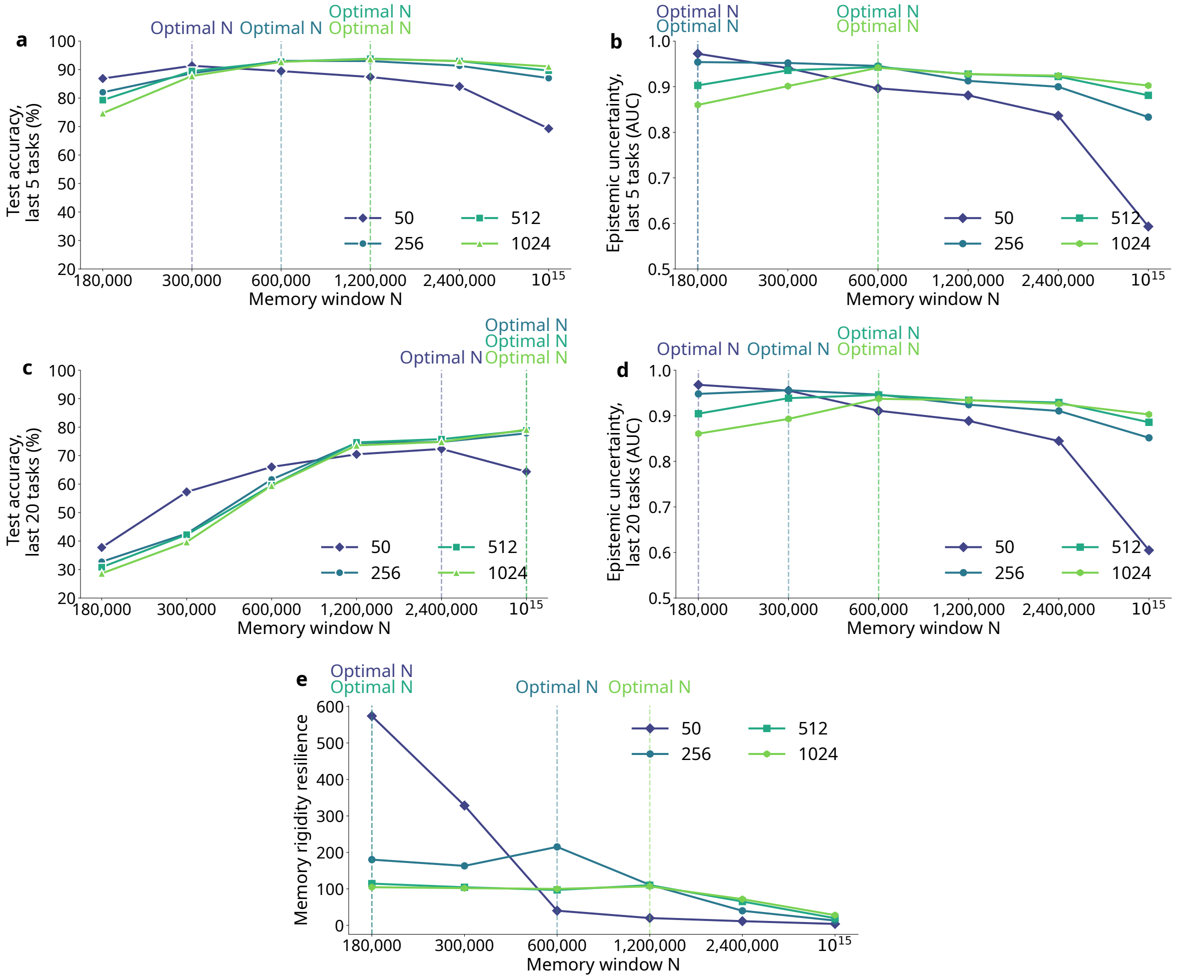

Supplementary Figure 3. Joint influence of N and model width. Performance metrics on 200-task Permuted MNIST for networks with 50–1024 hidden units. All results are presented for five finite memory windows and the unbounded case $N = 10^{15}$ that behaves like FOO-VB Diagonal. **a** Accuracy on the last five tasks. **b** Epistemic uncertainty (Fashion-MNIST, last five tasks). **c** Accuracy on the last 20 tasks. **d** Uncertainty, average over 20 tasks. **e** Memory-rigidity resilience. Lines guide the eye; all results are averaged over five runs.

shows the shorter-term (last five tasks) accuracy, and Supplementary Fig. 3c shows the longer-term (last 20 tasks) accuracy.

Two trends emerge from these results. First, the optimal N increases with model width. When optimizing for last-five-tasks accuracy, the 50-unit network favors $N = 300,000$, whereas the 512- and 1024-unit networks reach their best short-term accuracy at $N = 1,200,000$, no longer matching the expected value in terms of number of tasks. This suggests that when a network has higher capacity, it becomes more favorable to remember higher number of tasks. Second, beyond 512 units the marginal gain from further widening the network becomes negligible for short-term accuracy, although it still benefits long-term retention. These results confirm that N should scale with the number of parameters:

wider models can consolidate a larger window without sacrificing plasticity.

The same experiment also reveals a clear interaction between N and epistemic uncertainty, as seen in Supplementary Fig. 2b,d. Increasing network size allows preserving out-of-distribution data identification using epistemic uncertainty at higher N values, even when the network is experiencing catastrophic remembering.

To interpret these results, Supplementary Fig. 4 plots the distribution of synaptic variances at the end of training, in all situations considered in Supplementary Fig. 3. The results show that higher N values bring the synapses near zero, reflecting consolidation. In smaller, lower-capacity networks nearly all variances collapse towards zero when N increases, an unmistakable sign that every parameter has been pulled into the low-uncertainty regime and that the model has no degrees of freedom left for incoming tasks. This also explains why the network loses epistemic uncertainty capability: it becomes deterministic when attempting to train it with N values that exceed its capacity.

By contrast, the wider, high-capacity models keep a fraction of weights at high variance, i.e. in a still-plastic state, even when the window extends to twenty tasks. MESU therefore offers two complementary levers: one may enlarge the network to increase the pool of potentially plastic synapses, or enlarge N to decide how much of this pool is actually devoted to remembering past information. The optima identified in Supplementary Fig. 3 sit in situations where the distribution of variances remains bimodal: some weights highly certain, others still uncertain because this balance maximises both retention and adaptability.

Another take-away of Supplementary Figs. 2a-d is that the optimal N value depends on the priority metric: short term accuracy, long-term accuracy or epistemic uncertainty evaluation. It is essential to have a well-defined objective to unlock the full potential of MESU.

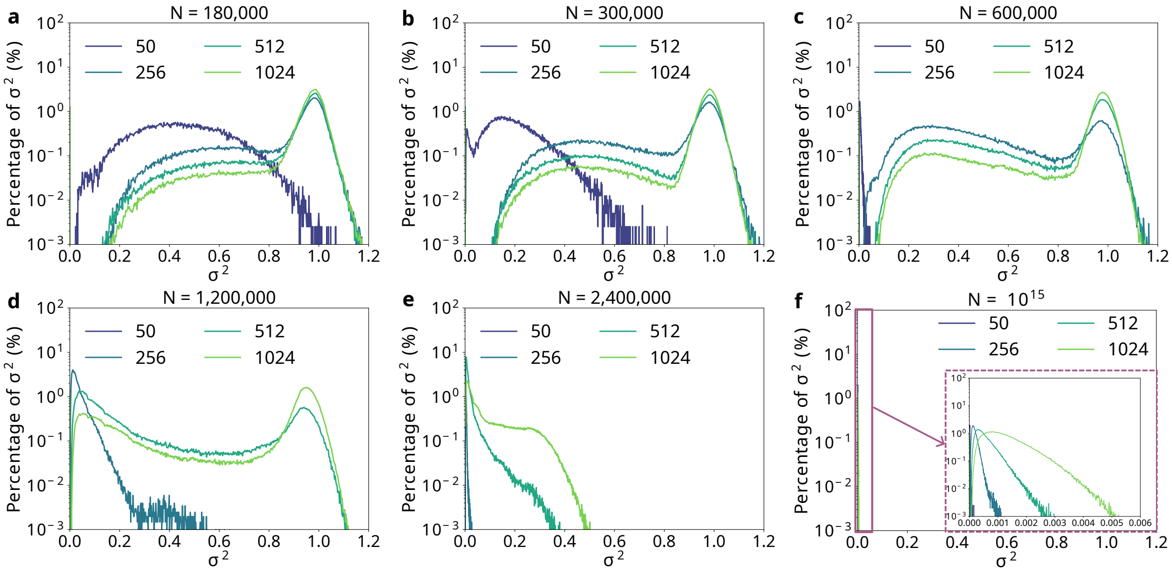

Supplementary Figure 4. End-of-training variance distributions. Histograms of σ^2 for networks with 50–1024 hidden units and memory windows increasing from three tasks (top left) to the unbounded regime (bottom right). Wider models tolerate larger N before all synapses consolidate.

Supplementary Note 7: Impact of the memory window N on the CIFAR-10 and CIFAR-100 datasets

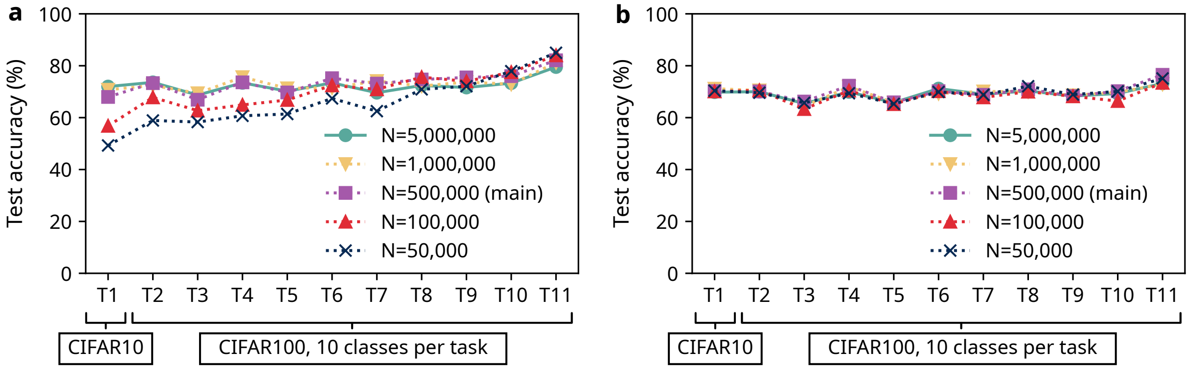

Supplementary Figure 5. Impact of the memory parameter N in MESU on task-incremental learning with CIFAR-10 and CIFAR-100. a Final accuracy on each of the 11 tasks in the single split case. **b** Same measure for the 16-splits case. Each curve corresponds to a different value of N; lines are guides for the eye.

In this Note, we investigate how the MESU memory window N influences performance on the CIFAR task-incremental benchmark. The network architecture (four convolutional layers plus two fully connected layers) and training protocol follow the main text: Task 1 is the ten-class CIFAR-10 dataset, Tasks 2–11 are ten class-groups from CIFAR-100, each task adds its own ten-unit head, while earlier layers are shared.

Supplementary Fig. 5a compares five values of N, ranging from $50,000$ to $5,000,000$, in the classical single-split setting. Larger windows ($N \geq 500,000$) recover higher accuracy on the oldest tasks, confirming stronger resistance to catastrophic forgetting. Conversely, the very latest task benefits marginally from smaller N, reflecting greater plasticity. Overall, $N = 500,000$, the value used in the main text, yields the best global balance, topping the average accuracy table (Supplementary Table 2). Note that the largest window in the sweep ($N = 5,000,000$) has negligible forgetting and reproduces, in practice, the behavior of FOO-VB Diagonal on this architecture.

We then adopt the 16-split protocol in which each task is divided into 16 equal subsets presented in round-robin order. Under this highly intermixed stream (Supplementary Fig. 5b) all N variants converge to nearly identical accuracies, indicating that only a modest memory window is required when successive tasks are tightly shuffled. This mirrors the observation in the main paper that even plain Adam matched or surpassed boundary-based continual learning techniques baselines (EWC and SI) under the same 16-split schedule (with MESU obtaining the highest accuracy).

Memory window N	Average test accuracy (%)	
	All 11 tasks	Last 10 tasks
**50,000**	65.85	67.51
**100,000**	70.31	71.66
**500,000 (main)**	**73.46**	**74.01**
**1,000,000**	73.19	73.43
**5,000,000**	72.52	72.57

Supplementary Table 2. Impact of the memory parameter N in MESU on task-incremental learning with CIFAR-10 and CIFAR-100. Averaged final accuracy on the 11 tasks and last 10 tasks in the single split case.

Supplementary Note 8: Inference time and accuracy

Category	Inference time (ms)	Last task accuracy (%)	Training memory occupation (MB)
**MESU**	0.1 ± 0.02	**94.50** ± 0.15	0.3
**FOO-VB Diagonal**	0.1 ± 0.02	68.80 ± 1.94	0.3
**SI**	**0.04** ± 0.01	91.09 ± 0.61	0.45
**EWC Online**	**0.04** ± 0.01	92.75 ± 0.29	0.45
**EWC Stream**	**0.04** ± 0.01	93.30 ± 0.43	0.45
**SGD Baseline**	**0.04** ± 0.01	93.10 ± 0.22	**0.15**

Supplementary Table 3. Comparison between Metaplasticity from Synaptic Uncertainty (MESU), Fixed-point Operator for Online Variational Bayes Diagonal (FOO-VB Diagonal), Elastic Weight Consolidation Online (EWC Online, which uses task boundaries), Elastic Weight Consolidation Stream (EWC Stream, which does not use task boundaries), Synaptic Intelligence (SI), and Stochastic Gradient Descent (SGD) in terms of inference time, accuracy, and memory occupation. Best results are reported in bold. Results for inference time are computed for each of the 200 tasks in the benchmark and the standard deviation is reported over the 200 tasks. Accuracy is computed on the last task after training and the standard deviation is reported over the five runs. Memory occupation is reported as the total memory used by the model during training. Results are averaged over five runs using a Nvidia RTX 3090, using Jax.

Supplementary Note 9: Comparison with Presynaptic Consolidation

This Note extends our empirical study to Presynaptic Consolidation (PC), a regularization method that modulates synaptic plasticity through stochastic presynaptic gating[5]. We reproduced the setup of the original PC paper on Permuted MNIST and evaluated MESU under the same conditions. We trained the reference architecture—a multilayer perceptron with two hidden layers of 200 ReLU neurons—on the canonical ten-task Permuted MNIST benchmark. Each task was presented for ten epochs with a batch size of 100. For MESU, we performed a grid-search over the memory window N, the learning rates α_μ and α_σ, and the prior standard deviation σ_{prior} (see Supplementary Table 5). All results are averaged over five independent runs.

As summarized in Supplementary Table 4, MESU attains 92.34% average accuracy, outperforming

Algorithm	Average test accuracy (%)	Per-task accuracy (%)				
**MESU N=5,800,000**	**92.46 ± 0.58**					
		**#1**	**#2**	**#3**	**#4**	**#5**
		95.76 ± 0.17	91.93 ± 1.28	88.30 ± 3.15	89.52 ± 0.53	91.48 ± 1.03
		**#6**	**#7**	**#8**	**#9**	**#10**
		91.77 ± 1.28	92.64 ± 0.24	93.56 ± 0.43	94.54 ± 0.53	95.07 ± 0.15
**Presynaptic Consolidation**	85.88 ± 0.72					
		**#1**	**#2**	**#3**	**#4**	**#5**
		80.32 ± 4.52	84.53 ± 3.19	82.74 ± 1.18	85.26 ± 2.16	86.06 ± 0.49
		**#6**	**#7**	**#8**	**#9**	**#10**
		86.24 ± 0.11	86.33 ± 0.99	88.13 ± 0.41	88.87 ± 0.38	90.27 ± 0.20

Supplementary Table 4. Comparison of Metaplasticity from Synaptic Uncertainties (MESU) and Presynaptic Consolidation. Reproduction of the Permuted MNIST experiment of[5], computing the average test accuracy over ten Permuted MNIST tasks, with neural networks of two hidden layers of 200 neurons trained for ten epochs with a batch size of 100. Results are averaged over three runs. Individual task accuracies (mean ± std) for both MESU and Presynaptic Consolidation are shown below to highlight the stability-plasticity trade-off.

PC by nearly seven percentage points. The seven-point gap underscores that MESU retains substantially more information from earlier tasks while still learning the later ones effectively. PC stabilizes weights by progressively lowering the probability that a presynaptic neuron is active. While elegant and energy-efficient, this mechanism often freezes many synapses after only a few epochs, leading to reduced plasticity for later tasks. MESU, in contrast, keeps all variances strictly positive, dynamically balancing consolidation against ongoing adaptation. The resulting network retains enough degrees of freedom to learn new permutations while guarding against catastrophic forgetting.

We also attempted to apply PC to the strict online setting of Fig. 3 in the main paper (single-sample updates, no task boundaries). Even after extensive tuning, the method remained far below MESU and could not reach competitive accuracy, confirming that its stochastic gating scheme is best suited to mini-batch regimes with distinct tasks. MESU, by design, operates successfully in both batch-wise and fully streaming scenarios.

**Hyper-parameter**	**Value**
Hidden units	200 - 200
Memory window N	5,800,000
Learning Rate α_μ	0.46
Learning Rate α_σ	1.58
Prior Mean μ_p	0
Prior Std Dev σ_p	0.02
Clamp	1
Number of samples ω	10

Supplementary Table 5. Hyper-parameter values for MESU experiment in Supplementary Tab. 4

Hidden units		128				
**Algorithm**	**Average test accuracy (%)**	**Per-task accuracy (%)**				
**MESU N=2,800,000**	**91.88 ± 0.35**					
		**#1**	**#2**	**#3**	**#4**	**#5**
		91.35 ± 1.68	90.79 ± 1.73	90.04 ± 2.42	89.56 ± 2.09	90.92 ± 1.73
		**#6**	**#7**	**#8**	**#9**	**#10**
		92.21 ± 0.55	92.75 ± 0.43	93.33 ± 0.42	93.74 ± 0.24	94.16 ± 0.23
**UCB**	91.44 ± 0.04	**NC for all tasks**				

Supplementary Table 6. Comparison of Metaplasticity from Synaptic Uncertainties (MESU) and Uncertainty-guided Continual Bayesian Neural Networks (UCB). Reproduction of the Permuted MNIST experiment of ref.[6], computing the average test accuracy over ten Permuted MNIST tasks, with neural networks of one hidden layer of 128 neurons (approx. 0.1M parameters). Neural networks were trained for 200 epochs with a batch size of 64. Results are averaged over three runs for UCB and ten runs for MESU. Individual task accuracies (mean ± std) for MESU are reported to present the stability-plasticity dilemma. UCB per-task results were not computed (NC).

Supplementary Note 10: Comparison with Uncertainty-guided Continual Bayesian Neural Networks (UCB)

In this Note, we compare MESU with Uncertainty-Guided Continual Bayesian learning (UCB), a Bayesian continual learning technique based on a heuristic[6], that rescales the learning rate of each weight proportionally to its standard deviation after each task. MESU, in contrast, derives coupled update rules for both mean and variance from a variational free-energy objective. The comparison below follows the experimental design of the UCB paper on ten-task Permuted-MNIST on a 100k-parameters neural network. For MESU, we conducted a grid-search over $N, \alpha_\mu, \alpha_\sigma$, and σ_{prior}; the chosen values are listed in Supplementary Table 7.

Supplementary Table 6 shows that MESU slightly outperforms UCB. We attribute the performance gap to the fact that MESU's update rules emerge directly from minimising a well-defined free-energy that balances learning and forgetting, whereas UCB's rule affects only the means and therefore cannot counteract the long-term collapse of variances that we identified as "vanishing uncertainty". The additional benchmark therefore reinforces our central claim: a principled treatment of synaptic uncertainty, rather than a purely heuristic rescaling, leads to more reliable continual-learning performance. We conjecture that, for longer task sequences (e.g. the 200-task bench in Fig. 3 of the main text), MESU would again pull ahead even at large model sizes. (We could not execute the original UCB code due to legacy dependencies).

The sample standard deviations of MESU and UCB differ. To assert the significance of our results, we use Welch's t-test to compare their empirical means. Specifically, we test the null hypothesis $H_0 : \mu_{\text{MESU}} = \mu_{\text{UCB}}$ against the two-sided alternative $H_a : \mu_{\text{MESU}} \neq \mu_{\text{UCB}}$ to assess whether the population means differ, and the one-sided alternative $H_a : \mu_{\text{MESU}} > \mu_{\text{UCB}}$ to assess whether MESU's mean is greater than UCB's mean.

From the data ($\bar{x}_1 = 91.88,\ s_1 = 0.35,\ n_1 = 10;\ \bar{x}_2 = 91.44,\ s_2 = 0.04,\ n_2 = 3$), the Welch t-statistic is $t \approx 3.8916$ with Welch–Satterthwaite degrees of freedom $\nu \approx 9.718$. For the two-sided test, the p-value is $p_{\text{two-tailed}} \approx 0.0032$ indicating strong evidence against H_0 in favor of the conclusion

that the population means differ. For the one-sided test in the direction $\mu_{\text{MESU}} > \mu_{\text{UCB}}$, the p-value is $p_{\text{one-tailed}} \approx 0.0016$ which provides strong evidence against the null hypothesis in favor of the claim that MESU's mean is greater than UCB's mean.

**Hyper-parameter**	**Value**
Hidden units	128
Memory window N	2,800,000
Learning Rate α_μ	0.007
Learning Rate α_σ	0.02
Prior Mean μ_p	0
Prior Std Dev σ_p	0.68
Clamp	1
Number of samples ω	10

Supplementary Table 7. Hyper-parameter values for MESU in Tab. 6

Supplementary Note 11: Detailed trade-off analysis of MESU and FOO-VB Diagonal

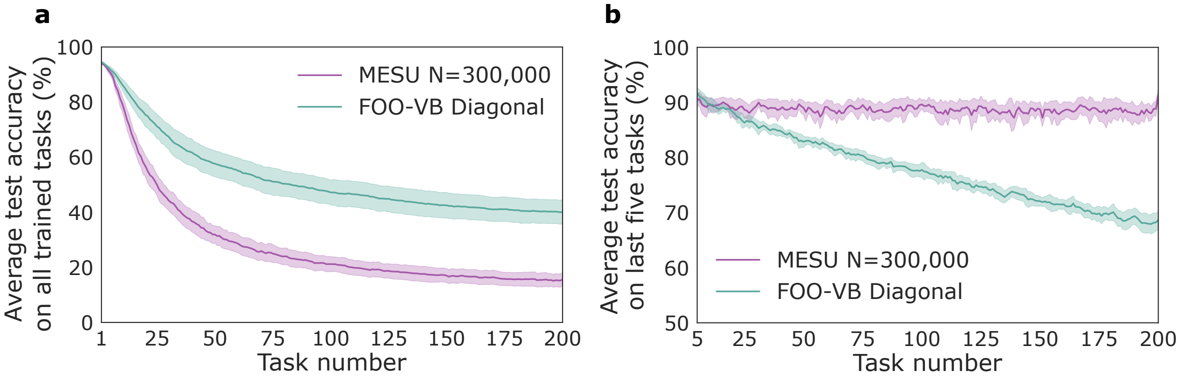

Supplementary Figure 6. Comparison of Metaplasticity from Synaptic Uncertainties (MESU) and Fixed-point Operator for Online Variational Bayes Diagonal (FOO-VB Diagonal) over 200 tasks of Permuted MNIST. a We display the average task accuracy on all previous tasks after training on a new task for both methods, and remark that FOO-VB Diagonal has largely superior average test accuracy than MESU due to having no forgetting mechanism. **b** We display the average test accuracy over the last five tasks after training on a new task for both methods. Conversely from a., if we restrain the average test accuracy to the last five tasks only, MESU capabilities remain constant whereas FOO-VB Diagonal drops significantly. Shading denotes one standard deviation over five runs.

This Note provides more elements on the accuracy of MESU and FOO-VB Diagonal training in the 200-tasks Permuted MNIST experiment (Fig. 3 of the main paper). Suppl. Fig. 6a shows the mean accuracy on all previously trained tasks, as a function of the number of trained tasks, throughout the 200-tasks Permuted MNIST training experiment. Mean accuracy is reported in both MESU and FOO-VB cases. Because FOO-VB never forgets, it preserves a higher global average over all 200

Permuted MNIST tasks: after the final task, its mean accuracy is 40.2±1.4%, whereas MESU, whose memory window N is five tasks, settles at 15.6±0.9%. The picture reverses when we focus on the tasks that remain inside that window (Suppl. Fig. 6b). MESU's accuracy on the five most recent permutations stays essentially constant around 90%, while FOO-VB falls to below 70% because its posterior variances collapse, learning rates vanish, and new information is absorbed poorly – an instance of catastrophic remembering.

Supplementary Note 12: MESU and Bayes by Backprop complexity

Bayesian neural networks inevitably require more computation than their deterministic counterparts because prediction and learning are performed by averaging over s Monte-Carlo (MC) weight samples. Each sample behaves as an independent model, so in the worst case training time grows linearly with s, i.e. $\mathcal{O}(s)$. Modern GPUs, however, process the s replicas in parallel, which limits the wall-clock increase. The same parallelism is not available for parameter tensors, hence memory consumption is expected to rise nearly one-for-one with s.

We compare the standard Bayes-by-Backprop (BBB) algorithm – where both the negative log-likelihood and the KL divergence between the variational distribution and the prior (a Gaussian mixture) are estimated with MC sampling – with MESU, which folds the KL term directly into the parameter update. Forward propagation and gradient back-propagation of activations are identical in both cases and at most s times slower than in a deterministic network. The difference appears when computing parameter gradients: BBB must also differentiate the KL term, whereas MESU does not. Consequently, the relative speed-up delivered by MESU depends on whether activation-gradient computation or parameter-gradient computation is the dominating cost. From a memory perspective, BBB is typically trained with Adam, whose two additional momentum buffers (for the mean and variance of the gradient) further increase the footprint, whereas MESU can be implemented with plain SGD-style buffers.

To quantify the practical impact we measured training time and peak GPU memory on an NVIDIA GeForce RTX 3090 for two convolutional networks trained on CIFAR-10 for ten epochs. The small model (Supplementary Fig. 7a,c) contains 1.25M parameters (2.5M when the mean μ and scale σ are stored separately) and processes roughly 1×10^5 activations per image per sample. The large model (Supplementary Fig.7b,d) doubles every channel width and enlarges the fully-connected layers, yielding 5M parameters (10M with μ and σ) and 2×10^5 activations.

When activation gradients dominate (small model), MESU and BBB display similar scaling with s (Supplementary Fig. 7a). Once parameter-gradient computation becomes the bottleneck (large model), BBB's extra KL term makes it noticeably slower, whereas MESU keeps the near-linear trend (Supplementary Fig. 7b). In both regimes, BBB consumes almost twice as much memory as MESU because of Adam's momentum buffers (Supplementary Fig. 7c,d).

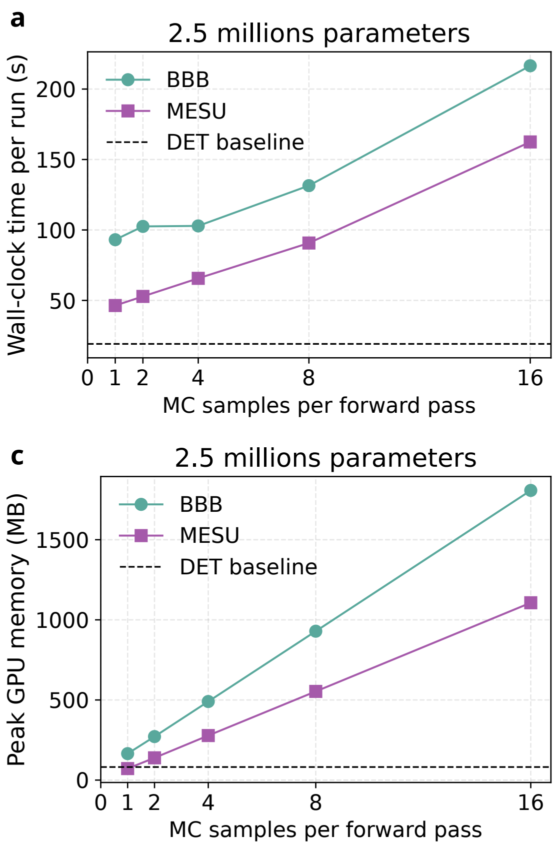

Supplementary Figure 7. Compute overhead of MESU and BBB. Wall-clock time per training run as a function of Monte-Carlo samples for (**a**) a 2.5 M-parameter CNN and (**b**) a 10 M-parameter CNN. Peak GPU memory per training run as a function of Monte-Carlo samples for (**c**) a 2.5 M-parameter CNN and (**d**) a 10 M-parameter CNN. MESU scales more favourably, with a shallower slope than BBB. The dashed line marks the deterministic baseline.

Supplementary References

1. Zeno, C., Golan, I., Hoffer, E. & Soudry, D. Task-agnostic continual learning using online variational bayes with fixed-point updates. *Neural Comput.* **33**, 3139–3177 (2021).

2. LeCun, Y. The mnist database of handwritten digits. *http://yann. lecun. com/exdb/mnist/* (1998).

3. Kirkpatrick, J., Pascanu, R., Rabinowitz, N., Veness, J., Desjardins, G., Rusu, A. A., Milan, K., Quan, J., Ramalho, T., Grabska-Barwinska, A. *et al.* Overcoming catastrophic forgetting in neural networks. *Proc. national academy sciences* **114**, 3521–3526 (2017).

4. Zenke, F., Poole, B. & Ganguli, S. Continual learning through synaptic intelligence. In *International conference on machine learning*, 3987–3995 (PMLR, 2017).

5. Schug, S., Benzing, F. & Steger, A. Presynaptic stochasticity improves energy efficiency and helps alleviate the stability-plasticity dilemma. *Elife* **10**, e69884 (2021).

6. Ebrahimi, S., Elhoseiny, M., Darrell, T. & Rohrbach, M. Uncertainty-guided continual learning with bayesian neural networks. *arXiv preprint arXiv:1906.02425* (2019).
